# Dynamics of Interaction among Professionals, Informal Supporters, and Family Caregivers of People with Dementia along the Dementia Care Pathway: A Nationwide Survey in Japan

**DOI:** 10.3390/ijerph20065044

**Published:** 2023-03-13

**Authors:** Hajime Takechi, Naoko Hara, Kyoko Eguchi, Shoko Inomata, Yuki Okura, Miwa Shibuya, Hiroshi Yoshino, Noriyuki Ogawa, Morio Suzuki

**Affiliations:** 1 Department of Geriatrics and Cognitive Disorders, School of Medicine, Fujita Health University, Toyoake 470-1192, Aichi, Japan; 2 Department of Gerontological Nursing, Niigata College of Nursing, 240 Shinnan-cho, Joetsu 943-0147, Niigata, Japan; 3 Faculty of Nursing, Shumei University, 1-1 Daigaku-cho, Yachiyo City 270-0003, Chiba, Japan; 4 Department of Nursing, Akita University Hospital, 44-2 Hasunuma Hiroomote, Akita-shi 010-8543, Akita, Japan; 5 School of Cultural and Social Studies, The Graduate University for Advanced Studies, Osaka 565-8511, Osaka, Japan; 6 Department of Occupational Therapy, Faculty of Health Sciences, Kyoto Tachibana University, 34 Oyakeyamada-cho, Yamashina-ku, Kyoto City 607-8175, Kyoto, Japan; 7 Alzheimer’s Association Japan, 811-3 Seimei-cho, Kamigyoku, Kyoto City 602-8222, Kyoto, Japan

**Keywords:** care pathway, dementia, family caregivers, national dementia strategy, support

## Abstract

This study aims to clarify the dynamics of information provision and human interaction to satisfy the needs of family caregivers. A questionnaire survey consisting of items on information received at and after diagnosis, persons and resources consulted, needs, and caregiver-oriented outcomes was conducted. Among the respondents, 2295 individuals who were caring for people with dementia were divided into quartiles by the time after diagnosis, and differences were statistically analyzed. The time after diagnosis in the first to fourth quartiles was 0.73 ± 0.4, 2.52 ± 0.49, 4.89 ± 0.73, and 10.82 ± 3.7 years, respectively. The number of persons consulted by family caregivers increased significantly from the first to the fourth quartiles (*p* < 0.001). During this time, attributes of professionals and informal supporters changed depending on the quartile. As time progressed, acceptance of the diagnosis increased, but so did its impact on the lives of family caregivers. These findings revealed differences over time in what family caregivers wanted and the dynamics of interactions that filled their needs. Informal supporters accounted for a significant proportion of the total resources. However, many family caregivers thought the information and support were insufficient. Thus, continuous reform of the care pathway is needed.

## 1. Introduction

The number of people with dementia is rapidly increasing with the aging of society, and it is estimated that this number will reach 74.7 million worldwide by 2030 and 131.5 million by 2050 [1]. In addition, the medical and social costs of dementia care were estimated to be USD 1 trillion in 2018, including the cost of directly caring for people with dementia and economic losses such as those resulting from family caregivers taking time off of work to provide care [1,2]. The number of people with dementia in Japan, the most aged society in the world, was estimated to be 4.6 million in 2012, and the societal costs of dementia in Japan were estimated at JPY 14.5 trillion in 2014 [3].

Life with dementia is a long-term process that can last more than a decade as it is a set of progressive conditions that last from onset until the end-of-life stage. Dementia gradually robs people of their autonomy and is difficult for those around them to understand [4,5,6]. Although it is known that good communication is important for family caregivers to relate positively to people with dementia, they are not always given enough opportunities to learn how to interact with their afflicted family member. In addition, it is necessary for professionals to provide not only education on how to communicate with people with dementia but also practical and psychological support to family caregivers regarding the difficulties they face in daily life [4,7,8,9].

Under these circumstances, national dementia strategies have been developed based on many past practices and studies. The dementia care pathway has been constructed to link care resources for people with dementia and their family caregivers [10,11,12,13,14].

However, although care pathways and national strategies have been designed and implemented, they are so multi-layered that their complexity may prevent family caregivers from accessing necessary information and support from local resources [15]. Therefore, it is important to clarify how family caregivers obtain the information they need and with whom they form relationships, as well as what processes they go through in relation to psychological acceptance and what life adjustments they make with respect to caregiving. To achieve this, we conducted a nationwide survey with the hypothesis that gaining a better understanding of the interaction dynamics communication among family caregivers and supporters along the care pathway would contribute to its further utilization and revision.

## 2. Materials and Methods

### 2.1. Participants and Procedures

The expert committee of the Alzheimer’s Association Japan (AAJ) planned the survey and mailed and collected the survey forms. To reach various family caregivers of people with dementia throughout Japan, the survey form was mailed to relevant places, including AAJ branches and members nationwide, prefectural and municipal governments, community comprehensive support centers, district medical associations, dementia specialists, medical centers for dementia disease, and dementia cafés. The total number of surveys mailed was 45,650. From each mailing address, family caregivers received the survey form and returned it in the attached envelope to the AAJ office. A QR code was attached to the survey form to enable response via the Internet. The survey was conducted from October to November 2021. Responses were received from all 47 prefectures in Japan, with 3514 total respondents. Family caregivers who were still caring for their loved ones were included in the present analysis, whereas those who had already completed end-of-life care (*n* = 729) were excluded. An additional 490 responses were excluded because of a lack of responses to survey items, i.e., those on family relationship (*n* = 116), living situation (*n* = 91), the age of the person with dementia (*n* = 65), the age of the family caregiver (*n* = 40), the place of receipt of the survey form (*n* = 19), and time after diagnosis (*n* = 159). Therefore, a final total of 2295 people were included in the analysis. This study was reviewed and approved by the AAJ ethics committee (No. 2021-011). The responses were anonymous, and it was clearly stated on the survey form that responding to the survey would be taken as an indication of consent to participate in the study.

### 2.2. Questionnaire

The survey form was discussed and prepared by the AAJ’s expert committee. The committee consisted of 16 members: two people with dementia, two family caregivers of people with dementia, one internist, one dementia specialist, four nurses, two occupational therapists, an AAJ representative, and three AAJ board members. Five of the members were also academic researchers. After two review meetings and additional e-mail exchanges, a survey form was developed.

The survey asked about the demographics of the people with dementia, including age, diagnosis of dementia or cognitive decline, living situation, length of time after diagnosis, whether they had applied for long-term care insurance (LTCI) and, if so, what the level of care needed was. Family caregivers of people with dementia were also asked about the following attributes: prefecture of residence, age, sex, relationship with the person with dementia, whether they lived with the person with dementia, employment status, and how they received the survey form. Regarding the level of care needed in the Japanese LTCI system, the certification for social care requirements is divided into eight levels according to the degree of cognitive and functional decline [16,17,18]: first, an independent level (no support from LTCI), followed by two support-needed levels at which little assistance is needed, and five subsequent care-needed levels at which more care is required. Social care services are offered based on the support- or care-needed level. Substantial numbers of people with dementia and their family caregivers do not wish to use social care services or to apply for certification. These participants typically have a mild level of cognitive decline and therefore, based on our previous study, were classified at the independent level in this study [17]. We use the term “Care Level” in this study instead of the terms “independent”, “support-need level”, and “care-need level” to present the levels on an ordinal scale. The independent level is regarded as Care Level 1. A relatively small number of people were certified as support-needed levels 1 and 2; therefore, in this study, these two levels were combined into one level and classified as Care Level 2. Then, the five care-needed levels were classified as Care Levels 3 to 7.

Next, the following eight questions were asked to examine information access by the family caregiver, support provided, and caregiver-oriented outcomes along the dementia care pathway. The first three questions regarded information and support provided, specifically (Q1) explanations and information obtained from medical institutions at diagnosis or thereafter, (Q2) persons with whom family caregivers consulted about dementia and their daily life at the time of diagnosis or thereafter, and (Q3) what assistance family caregivers were looking for from the persons they consulted. The next two questions regarded the evaluation of information and support by family caregivers, specifically (Q4) the extent of information and support obtained and (Q5) satisfaction with the level of information and support obtained. Finally, three questions were asked regarding caregiver-oriented outcomes, specifically (Q6) “To what degree have you accepted the diagnosis of dementia as a family caregiver?”, (Q7) “Are you sometimes harsh toward the person with dementia?”, and (Q8) “Has the diagnosis of dementia affected or changed your social activities, hobbies, or interests?”. For Q1, Q2, and Q3, multiple-choice items were provided, and the respondents were asked whether each of the items applied to them. Multiple responses were allowed. Items Q4, Q6, Q7, and Q8 were all assessed on a four-point Likert scale (ranging from 1 to 4). A higher point indicates a higher level of agreement. Q5 was rated on a visual analogue scale (from 0 to 10). Descriptive responses were also obtained for several other questions, such as ones regarding acceptance and consultation; however, these were not included in this analysis.

### 2.3. Statistics

For a basic analysis, descriptive statistics were used to demonstrate the mean, standard deviation, total number, and percentage (%). The participants were divided into quartiles according to the length of time after the diagnosis of the people with dementia for whom they were caring, and the groups were designated as quartiles 1, 2, 3, and 4. Differences in parameters among the groups, such as information acquired, consultations, and the psychological situation of caregivers, were compared using the Kruskal–Wallis test. Chi-squared tests were used for categorical variables. After the chi-squared tests, a residual analysis was performed to identify group differences. The level of significance was set at 0.05. IBM SPSS Statistics for Windows (version 27; IBM, Armonk, NY, USA) was used for all statistical analyses.

## 3. Results

Table 1 shows the basic attributes of the people with dementia and their family caregivers who participated in this study. The average age of the people with dementia was 81.3 years, and Alzheimer’s disease accounted for 67.7% of the dementia diagnoses. The most frequent Care Level was Care Level 3 (27.3%). The average age of the family caregiver was 64.5 years, and 70.6% were female. Those living with a person with dementia accounted for 64.8% of the respondents. Mothers were the most common recipient of caregiving (34.5%), followed by husbands (25.1%) and wives (16.9%). Almost the same number of respondents were currently working (49.0%) and retired or otherwise not working (49.1%).

To analyze the changes in the long course of dementia, we divided the respondents into four groups based on how much time had passed after the diagnosis. Table 2 shows a summary of changes in the responses to survey questions according to the time after the diagnosis. The care level showed a significant increase with the time after the diagnosis (*p* < 0.001). The average number of different types of information obtained from medical institutions at and after the diagnosis of dementia was 4.47 ± 2.77 out of 13 items, showing no difference according to the time after the diagnosis. On the other hand, the number of persons consulted after the diagnosis of dementia increased significantly with the time after diagnosis (*p* < 0.001). The number of matters that the respondents wanted to discuss with the persons they consulted also showed a gradual but significant increase (*p* = 0.028). No significant difference in the extent to which they obtained the information they wanted (*p* = 0.381 or in the degree to which they were satisfied with the information (*p* = 0.809) were found across time periods. The proportion of family members who accepted the diagnosis of dementia increased significantly with time (*p* < 0.001), and a small but significant decrease was seen in the incidence of being harsh with the people with dementia (*p* < 0.001). Family caregivers were significantly more likely to think that their own lives had changed over time (*p* < 0.001).

The Information obtained at the time of diagnosis and afterward and the consulted persons were examined in detail (Table 3 and Table 4). First, regarding the information obtained at the medical institution at and after diagnosis, more than 70% of the respondents replied that they had obtained information about the diagnosis of the disease and therapeutic medications for dementia, and 49.4%, or approximately half, replied that they had obtained information about the severity of dementia. On the other hand, less than 40% of the respondents were informed about the future prospect of dementia and precautions for daily life, 36.4% were informed about LTCI services, and 27.8% were informed about how to cope with and accept the disease of dementia. Fewer than 20% of the respondents reported having received information about opportunities to talk with other family caregivers, consultation phone calls, and dementia cafés (Table 3).

The number of people consulted increased with the time after a diagnosis, as shown in Table 2. However, with respect to the individuals family caregivers consulted with, differences were observed between those who sought consultations early after diagnosis and those who did so as time passed (Table 4). Early after diagnosis, family members were the most frequent people consulted with, as were community comprehensive support center staff; however, contact with care managers significantly increased, and they became the most frequently consulted persons with the passage of time after diagnosis (*p* < 0.001). In addition, the frequency of consultations with care staff from long-term care facilities, friends and acquaintances with caregiving experience, other caregiving family members, and dementia café staff increased over time (*p* < 0.001, *p* = 0.004, *p* < 0.001, and *p* = 0.008, respectively). Family doctors and dementia specialists were also consulted by approximately 30% of the respondents, but no significant change over time was observed after diagnosis.

The most common matters that the respondents wanted to discuss with the individuals they consulted consisted of advice on daily life and how to interact with people with dementia. These questions were the same across all quartiles (Table 5). In addition, approximately 50% of the respondents wanted to know how the symptoms of dementia would progress and develop in the future, and this number increased significantly with the progression of the disease (*p* = 0.028). Advice on the use of social resources also increased significantly with time (*p* = 0.011). Approximately one-third of the respondents simply wanted to be heard and understood, and another one-third simply wanted to express their caregiving difficulties; the latter increased significantly with time (*p* < 0.001).

## 4. Discussion

After a family member is diagnosed with dementia, the dynamics of the interaction with medical and care professionals and informal supporters along the care pathway are important in terms of what information and support the family caregivers receive, how they accept the disease, and how they rebuild their own lives over the long period of time that follows. In this study, a nationwide survey was conducted to clarify the current situation in Japan. The results showed that after a diagnosis of dementia, family caregivers receive basic information from medical institutions and, over time, information and support from various professionals and informal supporters, such as care managers, friends, and acquaintances who have experience as caregivers. The results also indicated the importance of emotional support and information for coping with dementia. The level of satisfaction with the information obtained was approximately 60%, indicating that although they were receiving information and support to some degree of satisfaction, many still thought it was insufficient.

Care for people with dementia and their family caregivers is not something that can be achieved only by medical interventions and care alone; it is important to build a comprehensive and coordinated care system that includes local governments and social organizations [8,9,10,14,19,20,21]. In response to the increase in the number of people with dementia, each country is summarizing and assessing the challenges of care and presenting a framework for care pathways as a national strategy [10,11,12,22]. Some countries employ a one-stop service, while others may have a variety of entry sites [10]. Studies have been reported on the individuals who perform coordination of care and the significance of this coordination, suggesting the importance of such coordinators [23,24,25,26,27]. In the present study, we observed a gradual transition of the individuals performing coordination from the staff of community comprehensive support centers to care managers along the course of dementia. As community comprehensive support centers serve as a general contact point for comprehensive community care in Japan [10] and are expected to serve as a link to care managers and community resources such as LTCI services, it is assumed that this transition is the result of the usual process in the Japanese social care system. It has been pointed out that the needs of people with dementia and their family caregivers differ at each stage of dementia, so it is important to continue to build educational and support systems in accordance with the stages of dementia [9,28,29,30,31,32,33,34,35,36,37].

It was reported that medical institutions provided information at the time of diagnosis and thereafter that mainly regarded the treatment, name, and severity of the disease, as well as a certain amount of information regarding daily living, future prospects, and the use of social care services [38,39,40,41]. Similar results were obtained in this study. Furthermore, in the present study, no change with time in the involvement of family doctors and specialists after diagnosis was seen, indicating that a certain level of support is continuously provided. However, previous reports pointed out that referrals from medical institutions to the Alzheimer’s Association, an important place for providing information and psychological support to caregivers, are inadequate [42,43,44]. The present study also showed that the direct provision of information on the Alzheimer’s Association or other self-help groups from medical institutions was low. It is assumed that family caregivers themselves may have received information about the AAJ through Internet searches or were referred to the AAJ through information provided by care managers or acquaintances. An interventional study on the significance of information provided by medical institutions was conducted, and further research is warranted in this area [39].

The present study showed that family doctors and specialists are involved in the same proportions during the course of dementia; however, it has been reported that family doctors and specialists play different roles [45]. Further study on this point will be necessary in the future.

In the present study, a shift was observed in the informal resources of consultation and support from close family members to friends or acquaintances with care experience and other family caregivers. Informal supporters accounted for a significant proportion of the total, indicating that they are an important resource supporting the care pathway. As a place to meet other family caregivers, gatherings held by each branch of the AAJ have been expanding since 1980, with branches in all 47 prefectures across the country holding monthly gatherings to provide such opportunities. Furthermore, since 2012, based on the Orange Plan and the New Orange Plan national dementia strategies in Japan, it has been recommended that municipalities nationwide establish dementia cafés. Accordingly, dementia cafés have been set up by the private sector and local governments [46]. The number of cafés has recently increased to more than 8000 locations nationwide. Dementia cafés were initiated in the Netherlands in 1997 as Alzheimer’s cafés [47] and have come to exist around the world [46,48,49,50,51]. They are expected to become places that support the activities of people with dementia and provide educational and psychological support to their families [48,49,50,51,52,53,54,55,56]. However, because dementia cafés are a relatively new system, future developments must be closely monitored.

In the present study, we also analyzed changes over time in the family caregivers’ satisfaction with the information they received, their perceptions, the harshness in their interactions with the care recipient with dementia, and changes in their lives. The majority of family caregivers were positive about their perception of the diagnosis of dementia, due in part to the increase in social resources and the development of dementia awareness. Although a certain percentage of family caregivers perceived themselves to be harsh to their care recipients with dementia, this decreased over time. Changes in their lives increased over time after diagnosis. Consistent with these changes, an increase in the need for consulted persons to understand the difficulties of caregiving was observed. Previous studies have shown that not only medical and social support but also information and emotional support are important for family caregivers, and the present results seem to support this [57,58,59,60,61]. The importance of considering the health and lifestyle needs of family caregivers was also pointed out. This is an indispensable element in the construction of the care pathway [62,63,64,65]. Although it would be important to consider other factors regarding the well-being of caregivers such as sense of coherence, burnout, resilience, and coping, we did not include such factors in this study [66,67,68]. It is important to investigate this in the future.

The present study has several limitations. First, the survey questionnaires were distributed to a broad range of targets and areas to obtain a wide range of responses. Therefore, it is highly likely that people who were willing to take such a survey would have responded. Considering that such persons may be more willing to look for persons to consult, it is necessary to be cautious when generalizing the results. In the future, it will be desirable to conduct a comprehensive survey by targeting specific areas and people. Second, the number of survey items was narrowed down in order to conduct a broad survey. Based on the results, it may be necessary to conduct a survey using a more comprehensive questionnaire in the future. Third, as the present survey was cross-sectional in nature, it is difficult to discuss causal relationships. In the future, it would be desirable to conduct a longitudinal analysis; for example, on the relationship between the information obtained and changes in daily life. Fourth, although the current study presented changes in the relationship with the support person and the content of support with the time after diagnosis in order to demonstrate the current situation of the dementia care pathway in Japan, the need for support may be influenced in a more complex way by the attributes and psychological state of the people with dementia and their family caregivers. Further detailed analyses and research will be important in the future.

## 5. Conclusions

While the major framework of the dementia care pathway was previously constructed, the findings of this study clarified the information and support obtained by family caregivers of people with dementia, what they want from the individuals they consult, and the changes they experience in their feelings and lives with the time after the diagnosis of their family members. As care for people with dementia and their family caregivers is promoted according to a national strategy, information and support seems to be improving. However, many aspects remain inadequate. Based on these findings, continuous reform of the care pathway is desirable so that support can be adequately provided as needed.

## Figures and Tables

**Table 1:** Characteristics of the participants.

People with Dementia		
	Mean	SD
Age (years)	81.4	8.7
Diagnosis	*n*	%
Alzheimer-type dementia	1528	67.7
Vascular dementia	96	4.3
Dementia with Lewy bodies	115	5.1
Frontotemporal dementia	94	4.2
Mixed dementia	49	2.2
Diagnosed with dementia	266	11.8
Mild cognitive impairment	64	2.8
Other type of dementia	37	1.6
Not diagnosed	9	0.4
Total	2258	100
Living arrangements	*n*	%
Living alone	279	12.3
Married couple household	905	39.9
Living with children	753	33.2
Other	332	14.6
Total	2269	100
Care certificate (Care level)	*n*	%
Care level 1	249	11.1
Care level 2	160	7.1
Care level 3	614	27.3
Care level 4	401	17.8
Care level 5	364	16.2
Care level 6	201	8.9
Care level 7	258	11.5
Total	2247	100
**Family Caregivers**		
	**Mean**	**SD**
Age (years)	64.5	11.9
Sex (female, %)	70.6	
Living situation	*n*	%
Living together	1487	64.8
Living nearby	658	28.7
Living far away	150	6.5
Total	2295	100
Relationship with person with dementia	*n*	%
Husband	577	25.1
Wife	387	16.9
Father	199	8.7
Mother	792	34.5
Father-in-law	48	2.1
Mother-in-law	186	8.1
Brothers or sisters	27	1.2
Grandparents	39	1.7
Children	2	0.1
Other	38	1.7
Total	2295	100
Work	*n*	%
Working	1111	49
Not working	1112	49.1
Other	43	1.9
Total	2266	100

**Table 2:** Information acquired, consultations sought by, and psychological situations of family caregivers according to time from diagnosis.

	Total (*n* = 2295)		Quartile 1 (*n* = 481)		Quartile 2 (*n* = 591)		Quartile 3 (*n* = 552)		Quartile 4 (*n* = 671)		
	Mean	SD	Mean	SD	Mean	SD	Mean	SD	Mean	SD	*p*
Time from diagnosis (years)	5.14	4.42	0.73	0.4	2.52 *	0.49	4.89 *	0.73	10.82 *	3.7	<0.001
Care certification (Care Level)	3.94	1.76	2.76	1.41	3.38 *	1.43	4.14 *	1.55	5.10 *	1.67	<0.001
Number of types of information provided from medical institution (0–13)	4.47	2.77	4.22	2.65	4.45	2.84	4.54	2.81	4.6	2.75	0.196
Number of persons consulted (0–14)	3.58	2.04	3.22	1.83	3.49	1.93	3.74 *	2.12	3.78 *	2.17	<0.001
Number of matters requested for consultation (0–9)	3.41	2.01	3.18	1.99	3.38	1.96	3.48 *	1.98	3.52 *	2.07	0.017
Extent of information obtained (1–4)	1.92	0.65	1.92	0.64	1.96	0.65	1.9	0.65	1.91	0.67	0.317
Satisfaction with information obtained (0–10)	6.37	2.23	6.4	2.33	6.34	2.2	6.44	2.12	6.32	2.28	0.894
Extent of acceptance (1–4)	3.34	0.79	3.09	0.86	3.27 *	0.78	3.40 *	0.77	3.53 *	0.7	<0.001
Extent of harshness (1–4)	2.24	0.85	2.3	0.85	2.31	0.83	2.27	0.86	2.09 *	0.85	<0.001
Extent of change in life (1–4)	2.96	0.88	2.76	0.9	2.85	0.85	3.02 *	0.84	3.16 *	0.86	<0.001

Abbreviations: SD—standard deviation. Kruskal–Wallis test was performed. Results of the post hoc analysis are shown only between quartile 1 and other quartiles (* *p* < 0.01).

**Table 3:** Information provided from medical institutions.

	*n*	%
Name of the disease	1848	80.5
Severity of dementia	1179	51.4
Drugs for the treatment of dementia	1722	75.0
Future prospects of dementia	888	38.7
Daily life precautions related to dementia	941	41.0
Contact information in case of emergency (e.g., when a caregiver’s family member is unwell or the person with dementia is missing or in trouble)	375	16.3
Information about driving while suffering from dementia	410	17.9
Information booklet on dementia	505	22.0
Information on long-term care insurance services	848	36.9
Advice on how to cope with, feel, and accept the disease of dementia	669	29.2
Notification of opportunities for discussion among family caregivers in the same position, such as gatherings through family associations	407	17.7
Information about the availability of dementia consultation calls	165	7.2
Information on dementia cafés	299	13.0

**Table 4:** Persons consulted at the time of diagnosis of dementia and afterwards.

	Total	Quartile 1	Quartile 2	Quartile 3	Quartile 4	*p*
	2275	478	586	546	665	
Family members, *n* (%)	1319 (58)	307 (64.2) ^†^	356 (60.8)	314 (57.5)	342 (51.4) *	<0.001
Primary care physician, *n* (%)	809 (35.6)	163 (34.1)	218 (37.2)	210 (38.5)	218 (32.8)	0.146
Dementia specialist, *n* (%)	693 (30.5)	139 (29.1)	175 (29.9)	156 (28.6)	223 (33.5)	0.22
Long-term care facility staff, *n* (%)	660 (29)	73 (15.3) *	153 (26.1)	188 (34.4) ^†^	246 (37.0) ^†^	<0.001
Care manager, *n* (%)	1421 (62.5	231 (48.3) *	359 (61.3)	373 (68.3) ^†^	458 (68.9) ^†^	<0.001
Community comprehensive support center staff, *n* (%)	825 (36.3)	206 (43.1) *	219 (37.4)	186 (34.1)	214 (32.2) ^†^	0.001
Nurses at medical institutions, *n* (%)	176 (7.7)	36 (7.5)	47 (8)	40 (7.3)	53 (8)	0.965
Community dementia care promoters, *n* (%)	209 (9.2)	39 (8.2)	55 (9.4)	55 (10.1)	60 (9)	0.76
Workplace colleagues of the person with dementia, *n* (%)	53 (2.3)	9 (1.9)	12 (2)	15 (2.7)	17 (2.6)	0.755
Workplace colleagues of caregiver, *n* (%)	275 (12.1)	67 (14)	80 (13.7)	60 (11)	68 (10.2)	0.121
Friends with caregiving experience, *n* (%)	777 (34.2)	144 (30.1) *	179 (30.5) *	211 (38.6) ^†^	243 (36.5)	0.004
Friends without caregiving experience, *n* (%)	138 (6.1)	27 (5.6)	37 (6.3)	34 (6.2)	40 (6)	0.971
Other family caregivers, *n* (%)	508 (22.3)	45 (9.4) *	93 (15.9) *	131 (24)	239 (35.9) ^†^	<0.001
Dementia café staff, *n* (%)	300 (13.2)	49 (10.3) *	64 (10.9)	82 (15)	105 (15.8) ^†^	0.008

Pearson’s chi-squared test with adjusted standardized residual analysis was performed (* <0.05 less or ^†^ <0.05 more than expected).

**Table 5:** Matters family caregivers were looking for from the persons they consulted.

	Total	Quartile 1	Quartile 2	Quartile 3	Quartile 4	*p*
	2221	462	572	537	650	
Advice regarding daily life (points to keep in mind, etc.), *n* (%)	1319 (59.4)	267 (57.8)	336 (58.7)	325 (60.5)	391 (60.2)	ns
Advice regarding communication with people with dementia, *n* (%)	1242 (55.9)	250 (54.1)	317 (55.4)	299 (55.7)	376 (57.8)	ns
Information on the treatment of dementia, *n* (%)	731 (32.9)	167 (36.1)	195 (34.1)	162 (30.2)	207 (31.8)	ns
Information on the symptoms and progression of dementia moving forward, *n* (%)	1147 (51.6)	215 (46.5) *	294 (51.4)	276 (51.4)	362 (55.7) ^†^	0.028
Contact information for emergencies (e.g., when a caregiver’s family member is unwell, when the person with dementia is missing or in trouble, etc.), *n* (%)	429 (19.3)	80 (17.3)	98 (17.1)	123 (22.9)	128 (19.7)	ns
Financial matters and household finances, *n* (%)	452 (20.4)	95 (20.6)	121 (21.2)	115 (21.4)	121 (18.6)	ns
Advice on the use of social resources (long-term care insurance services, family groups, dementia cafés, etc.), *n* (%)	949 (42.7)	181 (39.2)	222 (38.8) *	248 (46.2)	298 (45.8)	0.011
Just wanting to be listened to, *n* (%)	814 (36.7)	155 (33.5)	225 (39.3)	196 (36.5)	238 (36.6)	ns
Wanting to be understood and to convey the difficulties of caregiving, *n* (%)	710 (32)	114 (24.7) *	186 (32.5)	173 (32.2)	237 (36.5) ^†^	< 0.001

Pearson’s chi-squared test with adjusted standardized residual analysis was performed (* <0.05 less or ^†^ <0.05 more than expected). Abbreviations: ns—not significant.

## Data Availability

The data presented in this study are available upon request from the corresponding author. However, consideration from the ethics board would be required.
